# Nasal Catarrh

**Published:** 1880-10

**Authors:** 


### BIBLIOGRAPHICAL.

*Nasal Catarrh.*—By Prof. Martin Coomes, M. D. Publishers: Bradly & Gilbert, Louisville, Ky., 1880.

This work, the author announces in the preface, was reluctantly prepared at the request of some friends to meet the requirements of a large class of general practitioners remote from metropolitan cities and from specialism. The requirement has been ably met in the production of a comprehensive treatise which does justice to the subject. Commencing with the anatomy of the naso-pharyngeal organs, the method of examining, and the appliances necessary are fully described, together with local medication, constitutional medication, and the effects of climate. The different phases of affection are clearly and minutely shown, with the causes and treatment, rendering the work an important and useful contribution to medical literature.

